# Low-Quality Structural and Interaction Data Improves Binding Affinity Prediction via Random Forest

**DOI:** 10.3390/molecules200610947

**Published:** 2015-06-12

**Authors:** Hongjian Li, Kwong-Sak Leung, Man-Hon Wong, Pedro J. Ballester

**Affiliations:** 1 Department of Computer Science and Engineering, Chinese University of Hong Kong, Sha Tin, New Territories 999077, Hong Kong; E-Mails: jackyleehongjian@gmail.com (H.L.); ksleung@cse.cuhk.edu.hk (K.-S.L.); mhwong@cse.cuhk.edu.hk (M.-H.W.); 2 Cancer Research Center of Marseille, INSERM U1068, F-13009 Marseille, France; Institut Paoli-Calmettes, F-13009 Marseille, France; Aix-Marseille Université, F-13284 Marseille, France; and CNRS UMR7258, F-13009 Marseille, France

**Keywords:** docking, binding affinity prediction, machine-learning scoring functions

## Abstract

Docking scoring functions can be used to predict the strength of protein-ligand binding. It is widely believed that training a scoring function with low-quality data is detrimental for its predictive performance. Nevertheless, there is a surprising lack of systematic validation experiments in support of this hypothesis. In this study, we investigated to which extent training a scoring function with data containing low-quality structural and binding data is detrimental for predictive performance. We actually found that low-quality data is not only non-detrimental, but beneficial for the predictive performance of machine-learning scoring functions, though the improvement is less important than that coming from high-quality data. Furthermore, we observed that classical scoring functions are not able to effectively exploit data beyond an early threshold, regardless of its quality. This demonstrates that exploiting a larger data volume is more important for the performance of machine-learning scoring functions than restricting to a smaller set of higher data quality.

## 1. Introduction

The development of machine-learning scoring functions (SFs) for docking has become a vibrant research area. Machine-learning SFs employ non-parametric machine learning techniques to predict the binding affinity of a protein-ligand complex from a description of its structure. Such a description takes the form of a set of features, also called descriptors or energetic contribution terms, which are usually derived from an X-ray crystal structure. In contrast, classical SFs, either force-field, empirical or knowledge-based, assume a predetermined additive functional form relating structure and binding affinity, typically using Multiple Linear Regression (MLR).

There are now many machine-learning SFs, mostly designed to be applied to either virtual screening [1,2,3] or binding affinity prediction [4,5,6,7]. SFs for virtual screening aim at discriminating between binders and non-binders in large data sets of candidate molecules, whereas SFs for binding affinity prediction are designed to provide the most accurate estimate of the strength with which a molecule binds to a macromolecular target. The objective is, therefore, to design the most predictive SF for the intended application. Here we focus on the second application where the SF has to provide the best possible prediction of binding affinity, typically in terms of pKd or pKi, the negative logarithm of binding or inhibition constants. This SF is immediately useful to calculate a reliable estimate of the binding strength of protein-ligand complexes from their X-ray crystal structures, which would add value to structural interactomics databases [8,9,10]. Another use of accurate binding affinity prediction is in identifying the most potent chemical derivatives of a drug lead or fragment by ranking these molecules by their predicted affinity, thus reducing the time and financial costs that would be incurred by experimentally measuring the binding affinity of all the derivatives instead. SFs for binding affinity prediction can also be used to find those derivatives with less affinity for an off-target, which is helpful to design more selective chemical probes or reduce the side effects associated to an off-target. User-friendly re-scoring software implementing machine-learning SFs for these prospective applications is now freely available [11].

In constructing machine-learning SFs, we have favored the use of Random Forest (RF) [12] as the regression model, which, in our experience, has provided better results than Support Vector Regression (SVR) [13]. RF-Score [4] was the first machine-learning SF to achieve a substantial improvement over classical SFs at binding affinity prediction. RF-Score has been since then successful in prospective virtual screening [14], implemented in a large-scale docking web server called istar at http://istar.cse.cuhk.edu.hk/idock [15], and further improved [11,16,17].

It has been claimed elsewhere (e.g., [18]) that the quality of protein-ligand structures is essential to the development of methods for predicting binding affinity. There is, nevertheless, a surprising lack of systematic validation experiments in support of this hypothesis. Instead, such statements are often backed up with merely a few selected cases that agree with the presented hypothesis, an approach that is prone to confirmation bias. In a recent publication [17], the factors that contribute the most to predictive performance have been analyzed. One important finding is that a more precise chemical description of the complex does not generally lead to a more accurate prediction of binding affinity. This result is not so surprising once one reflects on the fact that a binding constant is experimentally determined in solution along a trajectory in the co-dependent conformational space of the interacting molecules, whereas the X-ray crystal structure just represents a possible final state of that dynamic process in a crystallized environment. Hence, there is no reason to think that very precise descriptors calculated from the crystal structure are necessarily more representative of the dynamics of the binding molecules than less precise descriptors. A corollary of this finding is that adding low-quality complexes to the training set, instead of being detrimental for the development of scoring functions as widely assumed, might actually be beneficial.

Consequently, this study investigates whether training a SF with data containing low-quality structures and binding measurements is detrimental or beneficial for predictive performance. We are adopting the widely-used quality criteria of the PDBbind database [19,20], where the refined set compiles high-quality complexes for the development and validation of SFs, and the general set also contains many more complexes of lower quality. The rest of the paper is organized as follows. Section 2 introduces models, data quality, benchmarks, and performance metrics. Section 3 discusses the results. Lastly, Section 4 presents the conclusions of this study.

## 2. Methods

### 2.1. Models

The four models that were evaluated and compared in a recent study [11] will be also used in this study to analyze new high-quality and low-quality training data. Model 1 is AutoDock Vina [21], which constitutes a demanding performance baseline given its condition as one of the best classical SFs at predicting binding affinity [11]. The only difference between models 1 and 2 is that the latter is recalibrated on each training set using Multiple Linear Regression (MLR). Thus, model 2 will allow us to observe the effect of training a classical SF with increasingly-sized data sets. The substitution of model 2’s MLR with RF regression gives rise to model 3. Likewise, model 3 will allow us to observe the effect of training a machine-learning SF with increasingly-sized data sets. Consequently, models 1–3 employ the same set of six features (five Vina energy terms and the number of rotatable bonds of the ligand). Lastly, model 4 only differs from model 3 in that it adds the 36 features from the original version of RF-Score [4] to the 6 Vina features used in models 1 to 3. Note that models 1 and 2 are classical SFs, whereas models 3 and 4 are machine-learning SFs.

Models 3–4 are stochastic and thus their performance on a test set has a slight dependence on the random seed with which RF training is carried out. To investigate this variability, for each RF model and training set, 10 random seeds were employed to give rise to 10 slightly different trained RF models. For each seed, we built a RF model using the default number of trees (500) and values of the mtry control parameter from 1 to 6 for model 3, and from 1 to 42 for model 4. The selected model for a given seed will be that with the mtry value providing the lowest RMSE (root mean square error, see Section 2.3) on the subset of training data known as the Out-of-Bag (OOB) data [12]. Thus, each combination of RF model and training set gives rise to 10 instances of the trained model with their corresponding predictions, *i.e.*, a boxplot of predictions. In this way, SFs can be compared by the median performance of their 10 RF model instances, which is a more accurate assessment than the usual procedure of basing the comparison on a single model instance from each SF.

This experimental setup was designed to address the following questions:

- How test set performance varies when Vina is tailored to the training set? (model 1 *vs.* model 2).
- How test set performance varies when a classical SF is converted into a machine-learning SF by substituting MLR with RF? (model 2 *vs.* model 3)
- How test set performance of a machine-learning SF varies with additional features? (model 3 *vs.* model 4)

Together with the increasingly-sized training sets that will be next introduced, these models explore the interplay of three major factors for performance: the regression model, the features and training data size.

### 2.2. Benchmarks

Four benchmarks were assembled from the PDBbind database. This database is commonly used to design and validate SFs for binding affinity prediction [19,22]. Only high-quality data is considered in the first three benchmarks, whereas the fourth also includes training data with lower degrees of quality.

#### 2.2.1. PDBbind Data Quality

We adopted the widely-used PDBbind definition of high-quality data for the development of scoring functions (high-quality complexes are placed in the PDBbind refined set) [19]. Here, a protein complex is considered to be of high-quality for the purpose of developing and validating scoring functions if all the following conditions are met:

(a) the resolution of the structure is better than 2.5 Å
(b) the structure has been determined by X-ray crystallography
(c) both the protein and the ligand are complete in the structure
(d) protein and ligand are non-covalently bound
(e) the ligand molecule does not contain uncommon elements such as Be, B, Si, and metal atoms
(f) in case of peptide ligands, these are oligo-peptides (*i.e.*, have less than 10 peptide residues)
(g) in case of nucleotide ligands, these are oligo-nucleotides (*i.e.*, have less than four nucleotide residues)
(h) binding data must be either a dissociation constant (Kd) or an inhibition constant (Ki)
(i) it is a binary complex (*i.e.*, those proteins with multiple ligands bound in close vicinity at the common binding site are discarded)

By definition, any complex that is not of high-quality is a low-quality complex (of course, the degree of quality of a complex within each group will be unavoidably different). The PDBbind general set adds low-quality complexes to those in the refined set. While we recognise that there are alternative definitions of what a high-quality complex is, some of these definitions are far more restrictive than the one we have adopted (e.g., [18]).

#### 2.2.2. Benchmarks Using High-Quality Data for Training

The first two benchmarks aimed at investigating the validity of previous results for machine-learning SFs in the light of newly released data. Specifically, the first benchmark consists in testing previously developed SFs, which were trained on the PDBbind v2013 refined set (*n* = 2959 complexes), on the PDBbind v2014 refined set minus v2013 refined set (*n* = 546). Therefore, this is a blind test in that only data available until 2013 was exclusively used to build the SF that predicts the binding affinities of the ‘future’ complexes that appeared in 2014. The second benchmark was recently constructed [11] to investigate how binding affinity prediction improves with training data size. This benchmark constructed the test set by comparing different time-stamped releases of the PDBbind database. In particular, the test set was the PDBbind v2013 refined set minus v2012 refined set (*n* = 382), and the four training sets of approximately equally incremental size were PDBbind v2002 refined set (*n* = 792), v2007 refined set (*n* = 1300), v2010 refined set (*n* = 2059) and v2012 refined set (*n* = 2897). Here, a fifth training set, PDBbind v2014 refined set minus the test set (*n* = 3076), is added to find out whether the conclusions of that study hold in the light of new data. By construction of each of these two blind benchmarks, the test set does not overlap with any of the training sets (this is also the case for the rest of benchmarks in this study). Furthermore, we had no control over the composition of training or test sets, as we either take all the complexes in the refined set or in the intersection of two database releases. It is worth noting that all test sets are naturally diverse, thus avoiding previous criticisms regarding the use of a test set connected by protein homology to the training set [22].

The third benchmark compares the resulting machine-learning SFs against a set of widely-used classical SFs. These were tested by others on the CASF-2013 [23], a updated version of the widely-used PDBbind benchmark [4] (this benchmark was introduced by Cheng *et al.* [19] but only recently named CASF-2007 [23]). CASF-2013 was chosen because it had been recently utilised to evaluate 20 SFs, mostly implemented in commercial software packages. The test set was PDBbind v2013 core set (*n* = 195) and the training set was PDBbind v2013 refined set minus v2013 core set (*n* = 2764). In this study, a second training set, PDBbind v2014 refined set minus v2013 core set (*n* = 3249), was included to examine how the predictive performance of the four models changes with new high-quality data.

#### 2.2.3. Benchmarks Including Low-Quality Data for Training

Finally, the fourth benchmark is intended to evaluate how SF performance depends on the volume and quality data. From the PDBbind v2012 general set, which consists of 9308 biomolecular complexes with different degrees of structural and interaction data quality, four filters were applied to remove 2234 non-protein-ligand complexes, 2 protein-ligand complexes that failed PDB-to-PDBQT conversion, 99 approximate binding affinity measurements (e.g., Kd ≤ 100 µM, Ki ~0.1 pm) and 92 NMR structures. After this initial filtering, 6881 X-ray crystal protein-ligand complexes were retained. We henceforth refer to this set as general12. From general12 complexes, we generated six training sets depending on interaction data quality (Kd/Ki or not) and structural data quality (three ranges in structure resolution). The largest training set is *general12* itself, including all filtered data regardless of its quality. The second training set is formed by the 6719 general12 complexes containing crystal structures with resolutions ≤3.0 Å (*general12* ≤ 3.0 Å), which discards 162 complexes with resolution between 3.0 Å and 4.6 Å. The third training set is made of the 5752 general12 complexes with resolutions ≤2.5 Å (*general12* ≤ 2.5 Å).

The remaining three training sets are constructed in an analogous way, but starting from the 4449 general12 complexes with Kd/Ki as interaction data (*i.e.*, discarding the 2432 complexes with IC50s, which constitute lowest-quality interaction data). This is the fourth training set of this benchmark (*general12_Kd/KiOnly*). Thereafter, the fifth training set is formed by the 4356 *general12_Kd/KiOnly* with resolutions ≤ 3.0 Å (*general12_Kd/KiOnly* ≤ 3.0 Å). Next, the sixth training set is made of the 3809 *general12_Kd/KiOnly* with resolutions ≤ 2.5 Å (*general12_Kd/KiOnly* ≤ 2.5 Å). Lastly, the seventh training set is the 2897 *refined12* complexes for comparison purposes. Note that complexes from both *general12_Kd/KiOnly* ≤ 2.5 Å and *refined12* have Kd/Ki only and resolutions ≤ 2.5 Å, but the former set contains 912 complexes that are of low quality because of not meeting at least of the other quality criteria (see Section 2.2.1).

The test set is the PDBbind v2013 refined set minus v2012 refined set (*n* = 382), the same set as in the second benchmark to allow comparison with SFs trained exclusively on high-quality complexes. There were no overlapping complexes between the test set and any of the seven training sets. Table 1 summarizes the training and test sets and the purpose of the four benchmarks. Note that refined14 is an abbreviation for PDBbind v2014 refined set. Other abbreviations can be similarly interpreted.

**Table 1:** Summary of the four benchmarks (number of complexes between brackets).

Benchmark	Test Set	Training Sets	Purpose
1	refined14\refined13 (546)	refined13 (2959)	To validate previous results for machine-learning SFs in the light of newly released data.
2	refined13\refined12 (382)	refined02 (792) refined07 (1300) refined10 (2059) refined12 (2897) refined14\(refined13\refined12) (3076)	To study how performance varies given more high-quality training data.
3	core13 (195)	refined13\core13 (2764) refined14\core13 (3249)	To compare machine-learning SFs to classical SFs on CASF-2013.
4	refined13\refined12 (382)	refined12 (2897) general12_Kd/KiOnly ≤ 2.5 Å (3809) general12_Kd/KiOnly ≤ 3.0 Å (4356) general12_Kd/KiOnly (4449) general12 ≤ 2.5 Å (5752) general12 ≤ 3.0 Å (6719) general12 (6881)	To investigate how performance changes when adding low-quality training data.

### 2.3. Performance Measures

Predictive performance was quantified through root mean square error RMSE, standard deviation SD in linear correlation, Pearson correlation coefficient R_p_ and Spearman correlation coefficient R_s_ between the measured and predicted binding affinities of the test set complexes. Specifically, for a given model f, $p^{\left(n\right)}=f\left({\vec{x}}^{\left(n\right)}\right)$ is the predicted binding affinity given the features ${\vec{x}}^{\left(n\right)}$ characterizing the *n*^th^ complex and the performance metrics are defined as:

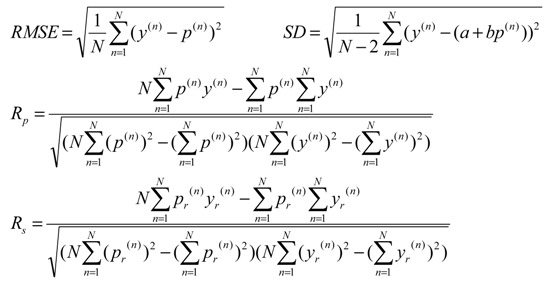
(1)
where *N* is the number of complexes in the set, a and b are the intercept and coefficient of the linear correlation between ${\left\{p^{\left(n\right)}\right\}}_{n=1}^{N}$ and ${\left\{y^{\left(n\right)}\right\}}_{n=1}^{N}$ on the test set, whereas ${\left\{p_{r}^{\left(n\right)}\right\}}_{n=1}^{N}$ and ${\left\{y_{r}^{\left(n\right)}\right\}}_{n=1}^{N}$ a are the rankings of ${\left\{p^{\left(n\right)}\right\}}_{n=1}^{N}$ and ${\left\{y^{\left(n\right)}\right\}}_{n=1}^{N}$, respectively.

Therefore, lower values in RMSE and SD and higher values in R_p_ and R_s_ indicate better predictive performance.

## 3. Results and Discussion

### 3.1. Evaluating RF-Score-v3 in a Blind Test on New Data Released in 2014

RF-Score-v3 [11], a new version of RF-Score [4], was recently built and validated. RF-Score-v3 is essentially RF::VinaElem trained on PDBbind v2013 refined set (*n* = 2959). The RF-Score-v3 software is freely available [11]. Since a new release of the PDBbind database is now available, we can further evaluate this software by testing RF-Score-v3 on this new data set (v2014 refined set minus v2013 refined set, *n* = 546). The results are RMSE = 1.39, SD = 1.39, R_p_ = 0.724, R_s_ = 0.717, whereas those of Vina are RMSE = 2.22, SD = 1.77, R_p_ = 0.481, R_s_ = 0.481. These results show that RF-Score-v3 exhibits remarkable performance on a blind test comprising such a diverse set of protein-ligand complexes.

### 3.2. Training with New High-Quality Data Stills Improves Predictive Performance

**Figure 1:**
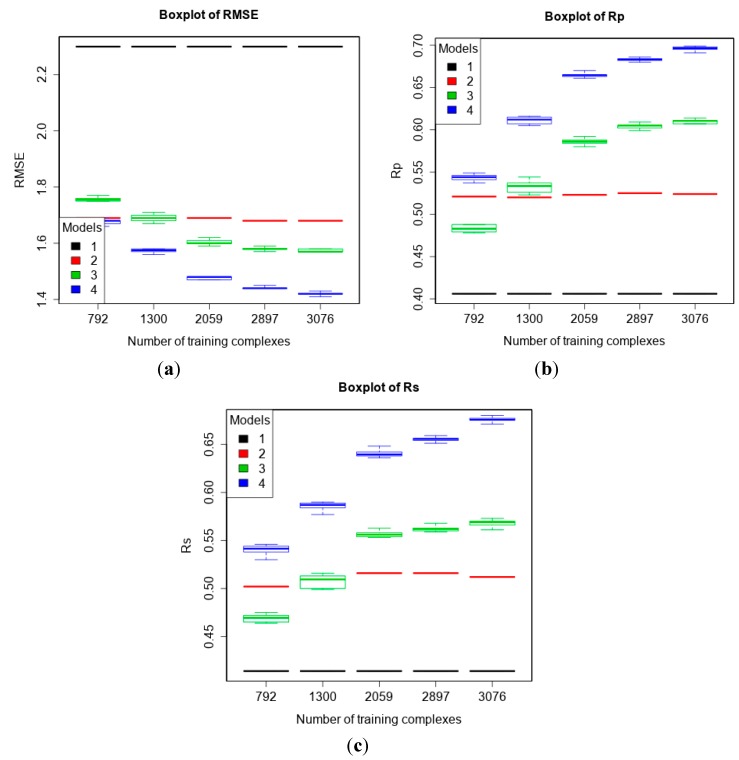
Performance of the four models with five training sets of incremental size on the test set of PDBbind v2013 refined set minus v2012 refined set (*n* = 382) according to RMSE (**a**); R_p_ (**b**) and R_s_ (**c**). Models: 1 (Vina), 2 (MLR::Vina), 3 (RF::Vina) and 4 (RF::VinaElem).

The PDBbind 2013 blind benchmark was introduced in a recent study [11]. Here, we used the same test set as well as the same four training sets comprising structural and interaction data up to a certain year. In addition, we added a larger training set comprising new high-quality data. The performances of the four models are shown in Figure 1 (Vina was executed off-the-shelf without re-training). It is observed that with more high-quality training data, the performance of MLR::Vina stayed nearly flat or even declined slightly, whereas the performance of RF::Vina and RF::VinaElem kept improving. As we anticipated [11], the performance gap between classical and machine-learning SFs increases with more training data. Notably, RF::VinaElem trained on the newly-added dataset of 3076 complexes led to a remarkable performance with RMSE = 1.41, SD = 1.41, R_p_ = 0.699, R_s_ = 0.680, the best results on this benchmark thus far.

**Figure 2:**
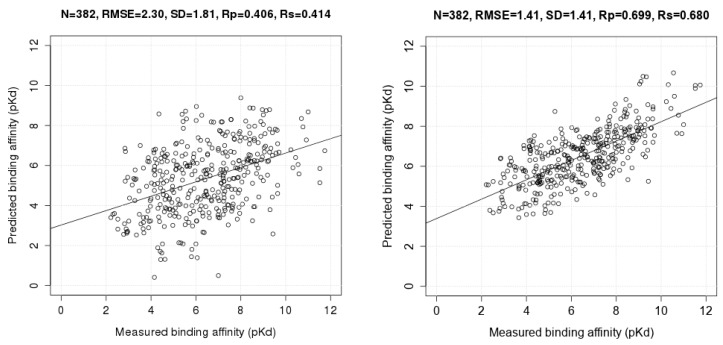
Correlation plots of measured and predicted binding affinities by AutoDock Vina (model 1; (**Left**)) and RF::VinaElem (model 2; (**Right**)) trained on the largest dataset comprising new high-quality data (*n* = 3076) tested on PDBbind v2013 refined set minus v2012 refined set (*n* = 382).

Figure 2 presents the two correlation plots comparing Vina and RF::VinaElem on this test set. Vina is one of the best classical SFs at binding affinity prediction [11] but its performance is substantially lower. See for instance the complexes with measured pKd of around 6 in Figure 2. For these complexes, Vina predicted a pKd between 1.7 and 9, whereas RF::VinaElem predicted the same complexes to have a pKd between 4.3 and 8, much closer to the measured affinity. Although there are some notable errors for each SF, overall the RMSE of RF::VinaElem is much lower than that of Vina, *i.e.*, 1.41 << 2.30.

### 3.3. Comparing Machine-Learning SFs to More Classical SFs on CASF-2013

The PDBbind benchmark [19] is a popular benchmark on which many SFs have been tested. Recently, an update of this benchmark has been introduced, the so-called CASF-2013 benchmark [20] for binding affinity prediction. Totally 20 SFs, e.g., X-Score, ChemScore, were benchmarked [23] on PDBbind v2013 core set (*n* = 195).

**Figure 3:**
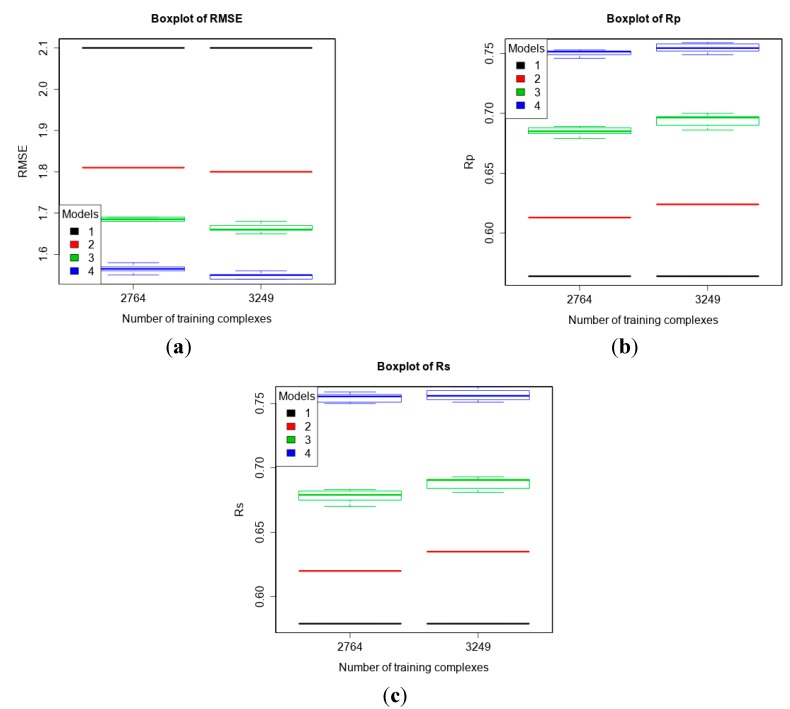
Performance of the four models trained on PDBbind v2013 refined set minus v2013 core set (*n* = 2764) and v2014 refined set minus v2013 core set (*n* = 3249) tested on v2013 core set (*n* = 195) according to RMSE (**a**), R_p_ (**b**), and R_s_ (**c**). Models: 1 (Vina), 2 (MLR::Vina), 3 (RF::Vina) and 4 (RF::VinaElem).

First, we evaluated the performance of the four models on PDBbind v2013 core set. The two training sets were v2013 refined set minus v2013 core set (*n* = 2764) and v2014 refined set minus v2013 core set (*n* = 3294). Figure 3 shows that more training data resulted in better predictive performance of machine-learning SFs, a conclusion consistent with previous studies [11,16]. RF::VinaElem achieved RMSE = 1.55, SD = 1.49, R_p_ = 0.752, R_s_ = 0.756 when trained on v2013 refined set minus v2013 core set (*n* = 2764), and RMSE = 1.54, SD = 1.47, R_p_ = 0.759, R_s_ = 0.763 when trained on v2014 refined set minus v2013 core set (*n* = 3294).

**Figure 4:**
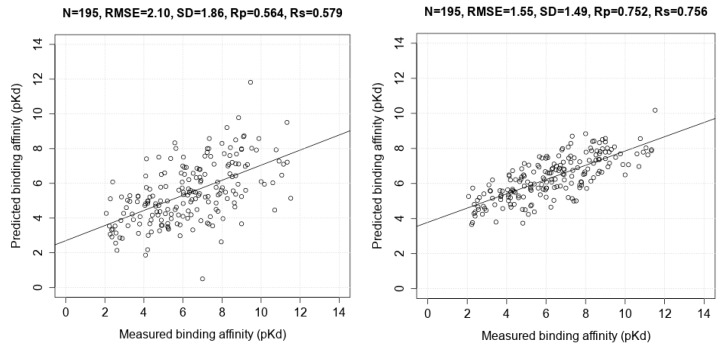
Correlation plots of measured and predicted binding affinity by AutoDock Vina (**Left**) and RF::VinaElem (**Right**) trained on PDBbind v2013 refined set minus v2013 core set (*n* = 2764) tested on v2013 core set (*n* = 195).

Figure 4 presents the two correlation plots comparing Vina and RF::VinaElem (model 4) trained with the 2764 complexes and tested on v2013 core set (*n* = 195).

Next, we compared the performance of Vina and RF::VinaElem to that of 20 other previously-tested classical SFs on the CASF-2013 benchmark by Li *et al.* [20] (Table 2). Some of these SFs were not able to score all the 195 test set complexes (this is not uncommon and is due to a range of reasons such as being unable to handle a particular atom type in the complex) and, thus, we provided this information by stating the number of test set complexes that were scored for each SF (N in Table 2). Table 2 shows that RF::VinaElem strongly outperformed all the previously-tested SFs, including those implemented in main-stream commercial software. The two baseline SFs by NHA (number of heavy atoms) and MWT (molecular weight) are first reported here in this study. The CASF-2013 benchmark paper [23] also reported ΔSAS, which is the buried solvent accessible surface area of the ligand molecule upon binding (*i.e.*, an estimation of the size of the protein-ligand binding interface).

**Table 2:** Performance of 22 scoring functions and 3 baselines on PDBbind v2013 core set (*n* = 195).

Scoring Function	N	R_p_	SD
RF::VinaElem	195	0.752	1.49
X-Score^HM^	195	0.614	1.78
ΔSAS	195	0.606	1.79
ChemScore@SYBYL	195	0.592	1.82
ChemPLP@GOLD	195	0.579	1.84
PLP1@DS	195	0.568	1.86
AutoDock Vina	195	0.564	1.86
G-Score@SYBYL	195	0.558	1.87
ASP@GOLD	195	0.556	1.88
ASE@MOE	195	0.544	1.89
ChemScore@GOLD	189	0.536	1.90
D-Score@SYBYL	195	0.526	1.92
Alpha-HB@MOE	195	0.511	1.94
LUDI3@DS	195	0.487	1.97
GoldScore@GOLD	189	0.483	1.97
Affinity-dG@MOE	195	0.482	1.98
NHA	195	0.478	1.98
MWT	195	0.473	1.99
LigScore2@DS	190	0.456	2.02
GlideScore-SP	169	0.452	2.03
Jain@DS	191	0.408	2.05
PMF@DS	194	0.364	2.11
GlideScore-XP	164	0.277	2.18
London-dG@MOE	195	0.242	2.19
PMF@SYBYL	191	0.221	2.20

### 3.4. Training with Low-Quality Data also Improves Predictive Performance

**Figure 5:**
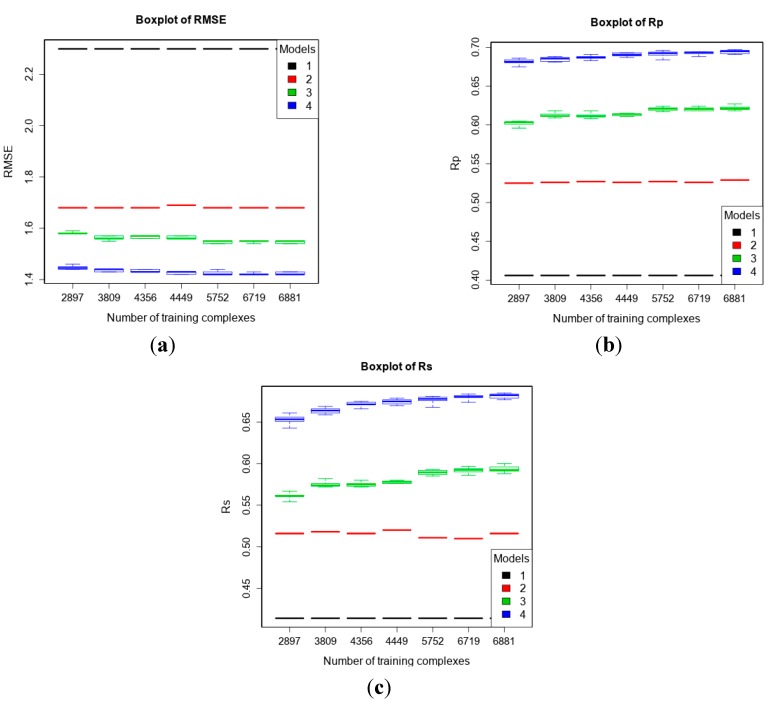
Performance of the four models trained with seven purposely-partitioned subsets of PDBbind v2012 general set on the test set of v2013 refined set minus v2012 refined set (*n* = 382) according to RMSE (**a**); R_p_ (**b**); and R_s_ (**c**). Models: 1 (Vina), 2 (MLR::Vina), 3 (RF::Vina) and 4 (RF::VinaElem).

We have established in a recent publication [24] that root mean square deviation RMSD generally has a small impact on binding affinity prediction, therefore there should be further gains by incorporating low-quality data for training. Again, as in the blind benchmark in Section 3.2, the test set was PDBbind v2013 refined set minus v2012 refined set (*n* = 382), and the training sets were six purposely-partitioned subsets of PDBbind v2012 general set according to the quality of structural and interaction data as explained in Section 2.2.1 as well as the v2012 refined set for comparison purposes. The results are shown in Figure 5. RF::VinaElem trained with 6881 complexes of both high- and low-quality achieved the best performance with RMSE = 1.42, SD = 1.42, R_p_ = 0.697, R_s_ = 0.684. These results are comparable to those obtained by the same SF trained with the newly-added dataset of 3076 high-quality structures (see right most column of results within each plot in Figure 1) and substantially better than those obtained by training on the 2897 high-quality complexes from the 2012 refined set only (see left most column of results within each plot in Figure 5). The latter demonstrate that adding low-quality structural and interaction data to the training set of a SF improve its performance.

**Figure 6:**
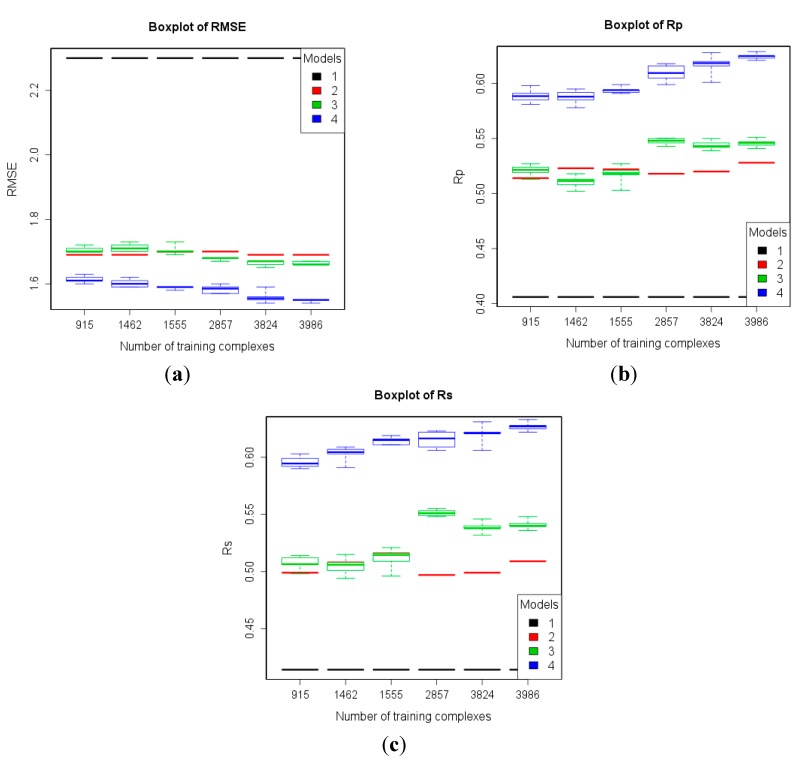
Performance of the four models trained with six subsets of PDBbind v2012 general set after subtracting refined12 on the test set of v2013 refined set minus v2012 refined set (*n* = 382) according to RMSE (**a**); R_p_ (**b**); and R_s_ (**c**). Models: 1 (Vina), 2 (MLR::Vina), 3 (RF::Vina) and 4 (RF::VinaElem).

These numerical experiments were repeated after subtracting the refined12 structures from each of the six training sets, which led to six new training sets of 915, 1462, 1555, 2857, 3824, and 3986 complexes. Thus, we analyze the level of performance that a SF can attain when trained exclusively on low-quality data (Figure 6), which is still quite high after removing the 2897 high-quality complexes. Again, the largest training set containing data of any quality leads to the best performance and the RF models still present an upward trend as more low-quality data is used for training, as it can observed in the results shown in the last and first column of each plot. We have also analyzed the impact of both types of data quality separately. In Figure 6, the test set performance from the sixth training set is significantly better than that from the fourth training set (3986 and 2857 training complexes, respectively), which means that adding training complexes with lower structural data quality (*i.e.*, resolution worse than 2.5 Å) improves test set performance. Likewise, the test set performance from the fourth training set is significantly better than that from the first training set, which means that adding training complexes with lower binding data quality (*i.e.*, IC_50_s) also improves test set performance.

## 4. Conclusions

We have presented comprehensive numerical experiments demonstrating that low-quality data is not only non-detrimental, but beneficial, for the predictive performance of scoring functions (SFs). This is true even when including in the training set complexes with resolution worse than 3 Å and IC_50_s instead of binding constants Kd/Ki, at least when having Random Forest as the regression model (we did not observe improvement with the linear regression model employed by classical SFs). However, the improvement coming from including low-quality data in the training set is smaller than that coming from high-quality data.

It is generally believed that removing low-quality data to focus on a training set of the highest quality is the best strategy to improve SFs. We have shown here that actually the opposite is true. The reason is that the quality of training data is not the only factor that affects SF performance, as data volume and diversity also play an important role. For instance, test set complexes can be outside the applicability domain of the model because of no similar high-quality data being included in the training set. In these cases, including similar low-quality training data should be better for performance than relying on the extrapolation of the model outside its applicability domain, as our results suggest.

As a byproduct of this study, we have also seen that a recently presented machine-learning SF, RF-Score-v3, achieves a very high performance on new test data, not available when this SF was constructed. Furthermore, new high-quality data for training continues to increase performance as anticipated. Lastly, we have shown that RF-Score-v3 performs substantially better than 20 classical SFs on the recently presented benchmark CASF-2013.
